# Regiospecific Methylation of a Dietary Flavonoid Scaffold Selectively Enhances IL-1β Production following Toll-like Receptor 2 Stimulation in THP-1 Monocytes*[Fn FN2]

**DOI:** 10.1074/jbc.M113.453514

**Published:** 2013-06-11

**Authors:** Eng-Kiat Lim, Paul J. Mitchell, Najmeeyah Brown, Rebecca A. Drummond, Gordon D. Brown, Paul M. Kaye, Dianna J. Bowles

**Affiliations:** From the ‡ Centre for Immunology and Infection, Hull York Medical School and Department of Biology and; ¶ Department of Biology, University of York, York YO10 5DD, United Kingdom and; the § Institute of Medical Sciences, University of Aberdeen, Aberdeen AB25 2ZD, United Kingdom

**Keywords:** Interleukin, Natural Products, Signaling, Small Molecules, Toll-like Receptors (TLR), IL-1β, Plant Natural Products, TLR Signaling, Methylated Flavonols

## Abstract

It is now recognized that innate immunity to intestinal microflora plays a significant role in mediating immune health, and modulation of microbial sensing may underpin the impact of plant natural products in the diet or when used as nutraceuticals. In this context, we have examined five classes of plant-derived flavonoids (flavonols, flavones, flavanones, catechins, and cyanidin) for their ability to regulate cytokine release induced by the Toll-like receptor 2 (TLR2) agonist Pam3CSK4. We found that the flavonols selectively co-stimulated IL-1β secretion but had no impact on the secretion of IL-6. Importantly, this costimulation of TLR2-induced cytokine secretion was dependent on regiospecific methylation of the flavonol scaffold with a rank order of quercetin-3,4′-dimethylether > quercetin-3-methylether > casticin. The mechanism underpinning this costimulation did not involve enhanced inflammasome activation. In contrast, the methylated flavonols enhanced IL-1β gene expression through transcriptional regulation, involving mechanisms that operate downstream of the initial NF-κB and STAT1 activation events. These studies demonstrate an exquisite level of control of scaffold bioactivity by regiospecific methylation, with important implications for understanding how natural products affect innate immunity and for their development as novel immunomodulators for clinical use.

## Teaser

**Background:** Plant natural products typically contain regiospecific modifications in their side chains that lead to different bioactivities.

**Results:** Only 3-*O*-methylated flavonols enhance IL-1β production in THP-1 cells costimulated with the Toll-like receptor 2 agonist Pam3CSK4.

**Conclusion:** Regiospecific methylation of flavonols controls their bioactivity as immunomodulators.

**Significance:** This study provides a platform to explore the use of regiospecific-modified natural products as novel immunomodulators.

## Introduction

There is increasing recognition of the beneficial properties of natural products for human health. Considerable attention has focused on the anti-oxidant properties of many plant-derived components of the diet, particularly the many small molecule products of plant metabolism (1, 2). Enhanced delivery of these nutraceuticals through fortification of food is one approach taken to improve health and wellness. In other examples, the small molecules are used directly as therapeutics or as the basis for the design of novel pharmaceuticals. In this context, there are a number of new applications in which the bioactivities of plant natural products are being used to affect cellular functions such as apoptosis and regulation of immune responses (3), as well as treat specific diseases such as those arising from viral, bacterial, and fungal infections and parasites including *Plasmodium* and *Leishmania* (4–6).

The commensal microflora of the gut plays a major role in regulating not only local mucosal immunity but also significantly, it affects systemic immune responses during health, autoimmunity, and infection (7, 8). Microbial sensing through specialized pattern recognition receptors of the Toll-like receptor (TLR),^3^ nucleotide-binding/oligomerization domain (NOD), and NOD-like receptor families is highly developed in macrophages, monocytes, and dendritic cells, and the ability of these cells to act as antigen presenting cells endows them with the capacity to translate microbial signals and thereby modulate both inflammation and long-lived acquired immunity (8, 9). Gram-positive intestinal bacteria are principally recognized through TLR2, but the response to bacterial TLR2 ligands such as lipoteichoic acid, peptidoglycan, and lipoproteins may be multifaceted. Thus, signaling through TLR2 may contribute to immune-mediated tissue dysfunction such as colitis while at the same time limiting bacterial replication (10, 11), assist in maintenance of the intestinal epithelial cell barrier, particularly in aged animals (12, 13), or be a factor contributing to invasive Gram-positive bacterial infections (14). Furthermore, TLR2 signaling has been reported to play a protective role in an experimental model of colitis-associated colorectal cancer (15), and polymorphisms of *TLR2* have shown to be associated with gastric cancer (16–18).

Several classes of plant natural products have been examined previously in the context of TLR2 signaling (19, 20), including one study addressing their role in colitis (21). However, the key feature of plant natural products is their extraordinary molecular diversity, arising from the plasticity of plant adaptive responses to their environment (22). A single scaffold structure can be decorated in many ways, whether through site-specific glycosylation, methylation, or countless other modifications, and the different products will typically exist as a complex mixture in plant extracts (23). This complexity has made it difficult to define the health benefits of any individual natural product in the context of TLR-mediated immune responses.

In this study, we have explored the activity of different scaffolds within the flavonoid family of natural products, and discovered that flavonols enhanced TLR2-induced IL-1β production with no effect on either IL-6 or TNF, two other major cytokines regulated by TLR signaling (24). Site-specific methylation of the flavonol scaffold was found to be critical for activity. The process did not involve inflammasome activation, but rather potentiation of IL-1β transcription, operating downstream of NF-κB. The results demonstrate how regiospecific methylation of defined scaffolds can alter cytokine profile and have broad implications for understanding the effects of natural products in the diet or when used as nutraceuticals.

## EXPERIMENTAL PROCEDURES

### 

#### 

##### Flavonoids

Quercetin, kaempferol, luteolin, eriodictyol, naringenin, hesperetin, catechins [(+)-catechin, (−)-epicatechin], and cyanidin were purchased from Sigma-Aldrich; fisetin, apigenin, 7,3′,4′-trihydroxyflavone, sakuranetin, isosakuranetin, quercetin-3-methylether, quercetin-7-methylether, quercetin-4′-methylether, 6-methoxyflavonol, 7-methoxyflavonol, quercetin-3,4′-dimethylther, kaempferol-3,7,4′-trimethylether, quercetin-3,7,3′,4′-tetramethylether were purchased from Extrasynthese (France); casticin was purchased from Chengdu Biopurify Phytochemicals Ltd (China).

##### THP-1 Culture and Stimulation

THP-1 cells were cultured in RPMI 1640 medium supplemented with 10% FCS, 2 mm l-glutamine, 100 unit/ml penicillin, 100 μg/ml streptomycin, and 50 μm 2-mercaptoethanol. To induce cytokine expression, 1 × 10^5^ cells were stimulated in a 200-μl volume with 25 ng/ml Pam3CSK4 (Autogen Bioclear) and various concentrations of flavonoids in a final concentration of 0.1% DMSO. The reactions were carried out in 96-well plates. After 24 h of incubation at 37 °C, the supernatants were collected for determination of secreted cytokines. For the time course study, the cells were stimulated in 24-well plates with modified conditions; each reaction contained 5 × 10^5^ cells, 25 ng/ml Pam3CSK4, and 10 μm flavonols in a 1-ml volume.

##### Cytokine Determination

The secreted IL-1β and IL-6 were detected simultaneously using BD CBA Flex Sets (BD Biosciences) following the manufacturer's instruction. The data were acquired using a CyAn ADP flow cytometer and analyzed with the software Summit version 4.3 (Beckman Coulter).

##### Western Blot Analysis

Cell lysates were extracted from 2 × 10^6^ cells with 100 μl 1× Denaturation Buffer of the BD CBA Cell Signaling Master Buffer Kit (BD Biosciences) and a mixture of phosphatase and protease inhibitors (Sigma-Aldrich). DNA in the lysates was degraded using protease-free DNase I (Roche). The lysates (50 μg) were clarified by centrifugation and separated on 12% SDS gels, transferred to 0.2 μm PVDF membranes and immunoblotted with anti-IL-1β antibody (Ab), anti-β-actin Ab (Sigma-Aldrich), anti-phospho-NF-κB p65(S536) Ab, anti-IκB-α Ab, anti-phospho-STAT1(S727) Ab, or anti-STAT1 Ab (Cell Signaling), followed by goat anti-rabbit or anti-mouse HRP-conjugated Ab (Santa Cruz Biotechnology), and were detected with the ECL Plus kit (GE Healthcare). For quantitation, the chemiluminescence films were scanned, and the images were analyzed using ImageJ.

##### Caspase-1 Activity Assay

The assay was carried out using Caspase-1/ICE Fluorometric Assay Kit (BioVision) in 96-well plates. Cell lysates were extracted from 2 × 10^6^ cells using cell lysis buffer provided by the kit. The lysates were diluted (1:20) for protein content measurement using BCA assay (Pierce). The lysates (50–200 μg) were then incubated with 50 μm YVAD-AFC substrate following the manufacturer's instruction. After 1 h of incubation at 37 °C, the samples were monitored in a BMG Labtech POLARstar OPTIMA microplate reader equipped with a 405 nm excitation filter and a 492 nm emission filter. As a positive control, the THP-1 cells were treated with 10 mm DTT at 37 °C for 1 h to induce caspase activity.

##### Real-time qPCR

Total RNA from 2 × 10^6^ cells was extracted using RNeasy Plus kit (Qiagen) and 1 μg of total RNA was reverse transcribed to cDNA by SuperScript® III reverse transcriptase (Invitrogen). Real-time qPCR was carried out in a ABI Prism 7000 system (Applied Biosystems); the reactions contained 20–50 ng of cDNA, Power SYBR Green PCR Master Mix (Applied Biosystems) and 10 μm primers specific to IL-1β (forward primer, 5′- CCACAGACCTTCCAGGAGAATG-3′; reverse primer, 5′-GTGCAGTTCAGTGATCGTACAGG-3′), TNF (forward primer, 5′-CTCTTCTGCCTGCTGCACTTTG-3′; reverse primer, 5′-ATGGGCTACAGGCTTGTCACTC-3′), GAPDH (forward primer, 5′- GTCTCCTCTGACTTCAACAGCG-3′; reverse primer, 5′-ACCACCCTGTTGCTGTAGCCAA-3′) or adenylyl cyclase-associated protein 1 (CAP-1) (forward primer, 5′-CAGTCTCTACCAGTTCAGGCTC-3′; reverse primer, 5′-ACTGGACCACTCTGAGCCTTCA-3′). The acquired data were analyzed using the comparative cycle threshold (C_t_) method of relative quantification (SDS software version 1.2.3, Applied Biosystems) to compare the levels of IL-1β in the stimulated cells to the untreated cells. The expression of IL-1β was normalized to the expression of a housekeeping gene GAPDH. A second housekeeping gene CAP-1 was used to validate the data normalization.

##### Detection of Phosphorylated MAPKs

Cell lysates were extracted from 2 × 10^6^ cells with 100 μl 1× Denaturation Buffer of the BD CBA Cell Signaling Master Buffer Kit (BD Biosciences). The samples were boiled for 5 min, centrifuged at 14,000 rpm for 5 min to remove the cell debris. Phosphorylated MAPKs (ERK1/2, JNK1/2, and p38) in the cell lysates were analyzed simultaneously using BD CBA Flex Sets (BD Biosciences) following the manufacturer's instruction. The data were acquired using a CyAn ADP flow cytometer and analyzed with the software Summit version 4.3 (Beckman Coulter).

##### Statistics

Comparisons of groups for statistical difference were carried out by Student's two-tailed *t* test.

## RESULTS

### 

#### 

##### Flavonols with Methylation at the C-3 Position Synergize with the TLR2 Agonist Pam3CSK4 to Enhance IL-1β Production

**FIGURE 1.**
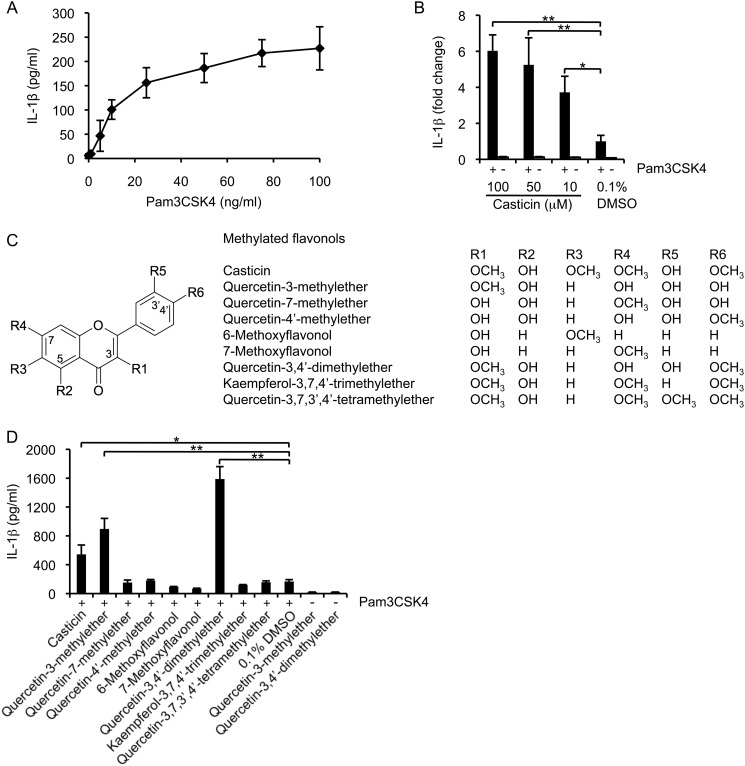
**Methylated flavonols enhance IL-1β secretion in Pam3CSK4-stimulated THP-1 cells.** *A*, THP-1 cells were stimulated with different amounts of Pam3CSK4. After 24 h of incubation, IL-1β levels were measured in supernatants. *B*, THP-1 cells were stimulated with casticin and 25 ng/ml Pam3CSK4 or with casticin alone. Cells treated with 0.1% DMSO were used as the control. Data are expressed as fold-change from cells treated with Pam3CSK4 alone. *C*, chemical structures of the methylated flavonols assayed in this study. *D*, IL-1β produced by THP-1 cells stimulated with Pam3CSK4 and 10 μm of each individual methylated flavonol. Data are expressed as the mean ± S.D. from three independent experiments. *, *p* < 0.05, **, *p* < 0.01.

The human monocytic cell line THP-1 was used to assess the ability of flavonoids to modulate cytokine secretion induced by the TLR2 agonist, Pam3CSK4, at a sub-optimal concentration of 25 ng/ml (Fig. 1*A*). In an initial screen, we examined 14 representative molecules from 5 flavonoid subclasses (supplemental Fig. S1) and assayed their effects at a range of concentrations on IL-1β and IL-6 production in the presence or absence of Pam3CSK4 (supplemental Fig. S2). Of these diverse structures, casticin was found to have a significant bioactivity. The effect was dose-dependent, was observed only in the presence of the TLR2 agonist and was selective in that the production of IL-1β was enhanced with no effect on IL-6 secretion (Fig. 1*B*, supplemental Fig. S2). A major distinction between casticin and three other closely related flavonoids that displayed only minimal effect on IL-1β secretion (quercetin, kaempferol, and fisetin), was the presence of methylation on the scaffold (supplemental Fig. S1). When the requirement for methylation was explored further, the presence and position of methoxy groups were indeed found to be critically important for the activity observed (Fig. 1, *C* and *D*). Casticin has four methoxy groups at the C-3, -6, -7, and -4′ positions. When additional flavonols were assayed, a single methylation at the C-3 position in quercetin-3-methylether was sufficient to confer activity. The greatest effect was seen with quercetin-3,4′-dimethylether. Further methylations at other positions reduced or abolished activity (Fig. 1*D*). In all cases, the impact of these flavonols on IL-1β secretion by THP-1 cells was only observed in the presence of the TLR agonist. These data demonstrate for the first time that regiospecific methylation of a natural product scaffold determines its capacity to affect cytokine secretion induced through the TLR2 signaling pathway.

##### 3-O-Methylated Flavonols Do Not Increase Caspase-1 Activity

Optimal IL-1β secretion requires the induction of gene transcription, often downstream of TLR signaling, together with caspase-1-dependent cleavage of the cytokine precursor protein, proIL-1β. Caspase-1 activity in turn is regulated by the inflammasome, a multiprotein complex activated through a variety of signaling and stress-related pathways (25). It was of interest therefore to determine whether the ability of the 3-*O*-methylated flavonols to enhance IL-1β secretion was reflected in an up-regulation of caspase-1 activity.

**FIGURE 2.**
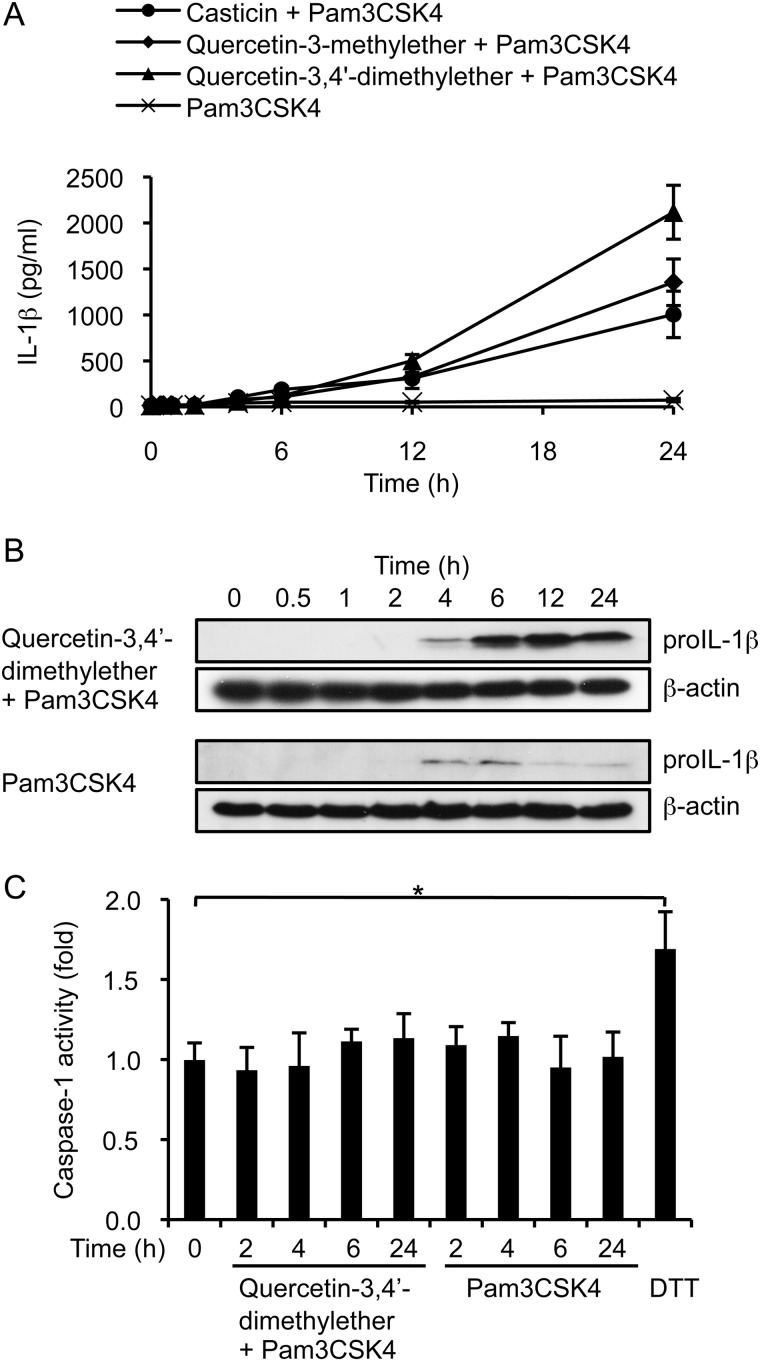
**3-*O*-Methylated flavonols do not increase caspase-1 activity in THP-1 cells.** *A*, levels of IL-1β secreted into culture media by cells stimulated with Pam3CSK4 and 10 μm methylated flavonol. *B*, Western blot analysis of proIL-1β levels in cell extracts after stimulation. β-Actin was used as the loading control. *C*, caspase-1 activity in cell extracts after stimulation. Fold-change in caspase-1 activity was determined by comparing the level found in stimulated cells with those of non-stimulated cells. Cells treated with 10 mm DTT at 37 °C for 1 h were used as a positive control. Data in *A* and *C* are expressed as the mean ± S.D. from three independent experiments. *, *p* < 0.01.

Kinetic analysis of IL-1β production following stimulation of THP-1 cells with Pam3CSK4 alone, or in combination with each of the three 3-*O*-methylated flavonols, indicated that the synergistic effects of the flavonols on IL-1β secretion were evident by 4 h post-stimulation and persisted up to 24 h, the final time point assayed (Fig. 2*A*). Western blot analysis of cell extracts harvested at the same time points showed that costimulation was necessary to elevate levels of proIL-1β (Fig. 2). In the extracts of cells treated with quercetin-3,4′-dimethylether and Pam3CSK4, proIL-1β was detectable by 4 h and increased in amount with time (Fig. 2*B*, *first row*). In contrast, in those extracts from cells treated with Pam3CSK4 alone, the precursor was only weakly and transiently present (Fig. 2*B*, *third row*).

Given that the synergistic effect of quercetin-3,4′-dimethylether and Pam3CSK4 was reflected both in IL-1β secretion and in the accumulation of the IL-1β precursor protein, we anticipated that there might also be an effect on the activity of caspase-1. However, caspase-1 activity was found to be near identical in cells treated with Pam3CSK4 alone and in those that had been costimulated (Fig. 2*C*). Together, these data indicate that the synergistic effect of methylated flavonols and Pam3CSK4 on secretion of IL-1β was not due to enhanced caspase-1 activity, but rather to an increased amount of IL-1β precursor that was available for processing by housekeeping levels of caspase-1.

##### 3-O-Methylated Flavonols Do Not Enhance Steady-state IL-1β mRNA Levels during the Early Responses of THP-1 Cells to the TLR Agonist

**FIGURE 3.**
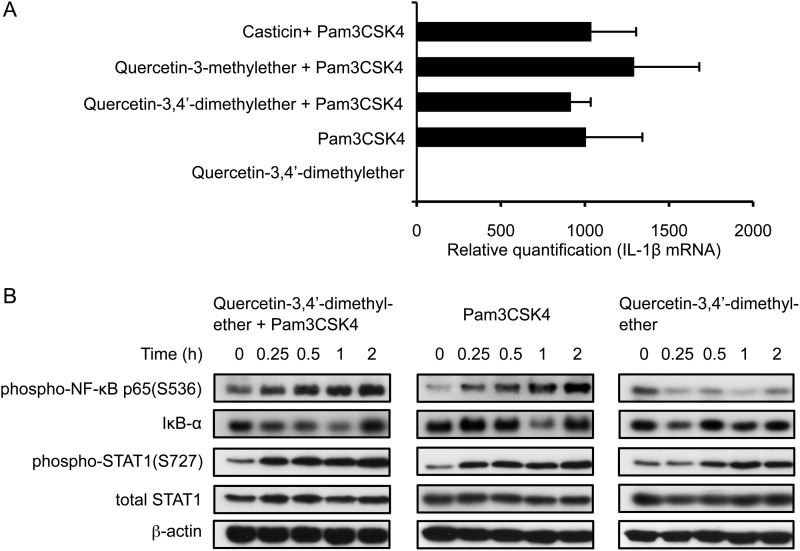
**Methylated flavonols do not affect steady state levels of IL-1β mRNA and associated transcriptional regulators within the first 2 h of stimulation of THP-1 cells.** *A*, real-time qPCR analysis of steady-state IL-1β mRNA levels in cells stimulated with Pam3CSK4 alone or costimulated with 10 μm methylated flavonol for 2 h. *B*, time course analyses of phospho-NF-κB p65(S536), IκB-α, phospho-STAT1 (S727), and total STAT1 in stimulated cells. Target proteins were detected on Western blots using specific Ab. β-Actin was used as the loading control.

Since costimulation of THP-1 cells with Pam3CSK4 and methylated flavonols led to an increased amount of proIL-1β precursor, we next analyzed changes in steady-state IL-1β mRNA levels in cells 2 h postexposure to these stimuli. Treatment with the TLR agonist alone, or costimulation with the methylated flavonols led to a near-identical induction of IL-1β mRNA. In contrast, quercetin-3,4′-dimethylether alone had no capacity to induce IL-1β mRNA (Fig. 3*A*).

We then examined the activation of NF-κB and STAT1, transcription factors known to be phosphorylated during TLR2 signaling and involved in IL-1β gene transcription (24, 26). Addition of quercetin-3,4′-dimethylether alone to the THP-1 cells did not activate NF-κB. In contrast, Pam3CSK4, or Pam3CSK4 in conjunction with methylated flavonol, led to increased levels of phospho-p65 within 30 min, and at 2 h ∼2.6-fold increments were observed in both samples (Fig. 3*B*, *first row*). A reduction in levels of the NF-κB repressor IκB-α was also observed at 1 h in those cells that had been Pam3CSK4-stimulated (1.6-fold reduction) or costimulated (1.4-fold reduction) (Fig. 3*B*, *second row*). Thus, Pam3CSK4-stimulation and costimulation both resulted in similar profiles of phospho-p65 and IκB-α. Under these two conditions of stimulation, STAT1 was activated as early as 15 min, whereas quercetin-3,4′-dimethylether alone induced a measurable but delayed increase in STAT1 phosphorylation (Fig. 3*B*, *third row*).

**FIGURE 4.**
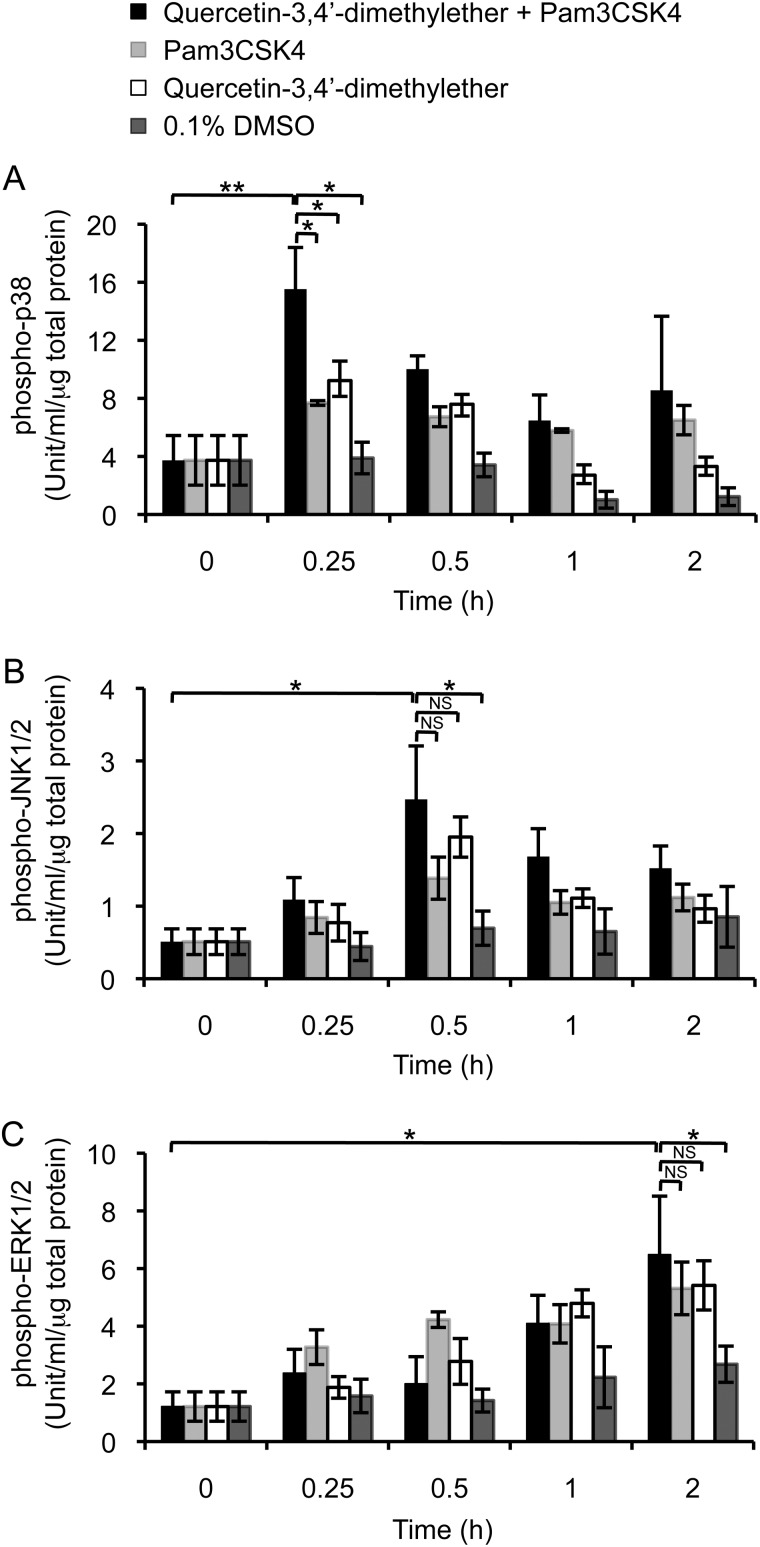
**Phosphorylation of MAPKs in THP-1 cells.** Levels of phosphorylated p38 (*A*), JNK1/2 (*B*), and ERK1/2 (*C*) in THP-1 cells incubated with Pam3CSK4 and/or 10 μm quercetin-3,4′-dimethylether. Data are expressed as the mean ± S.D. from three independent experiments. *NS*, not significant, *, *p* < 0.05, **, *p* < 0.01.

The activated p38 MAPK kinase is known to phosphorylate STAT1 at Ser-727 (27). We observed that application of either Pam3CSK4 or quercetin-3,4′-dimethylether led to increased levels in phospho-p38 peaking at 15 min post-stimulation (Fig. 4*A*). Under conditions of costimulation with Pam3CSK4 and methylated flavonol, the effect on levels of phospho-p38 was additive, suggesting the involvement of both TLR-dependent and TLR-independent signaling pathways. Analyzing other kinases, we found that under these conditions of costimulation, the timing of phosphorylation of JNK1/2 lagged behind that of p38, with phosphorylation of ERK1/2 occurring even later (Fig. 4, *B* and *C*). In contrast to the phosphorylation of p38 however, there was no additive effect on the phosphorylation of JNK1/2 and ERK1/2 under conditions of costimulation.

Taken together, stimulation with Pam3CSK4 alone or costimulation with the methylated flavonol for 2 h, resulted in similarly increased levels of steady-state IL-1β mRNA, a finding reinforced by the phosphorylation profiles of the transcription initiation factor NF-κB.

##### Methylated Flavonols Have Late Acting Effects on Steady-state IL-1β mRNA Accumulation

**FIGURE 5.**
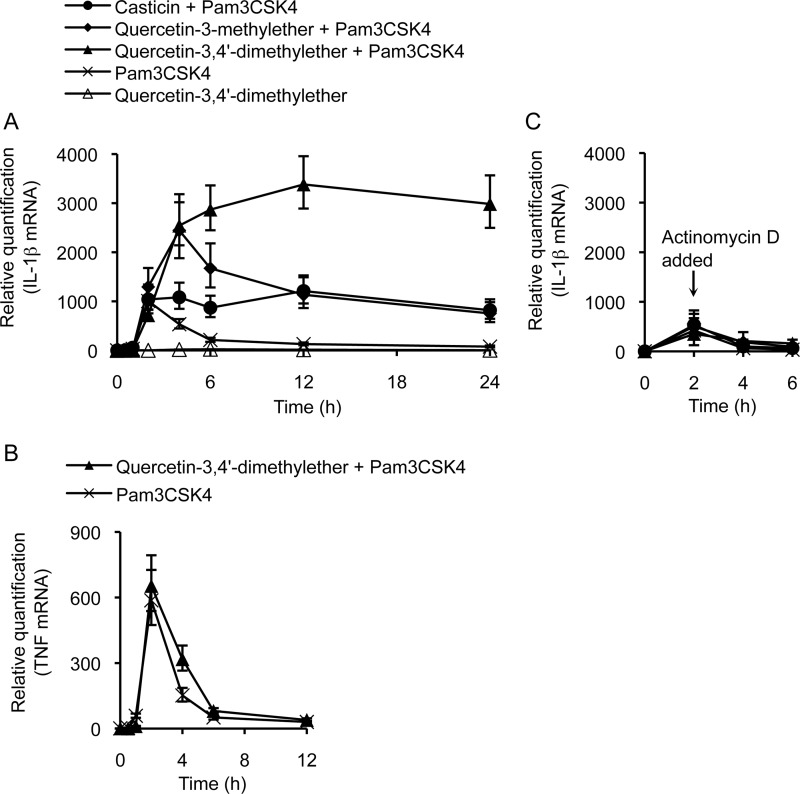
**Methylated flavonols lead to elevated levels of IL-1β mRNA after 2 h of stimulation of THP-1 cells.** *A*, real-time qPCR analysis of steady-state IL-1β mRNA levels in cells stimulated with Pam3CSK4 alone or costimulated with 10 μm methylated flavonols over time. *B*, real-time qPCR analysis of steady-state TNF mRNA levels in cells stimulated with Pam3CSK4 alone or costimulated with 10 μm quercetin-3,4′-dimethylether showing that quercetin-3,4′-dimethylether does not affect steady state levels of TNF mRNA in the stimulated cells. *C*, cells were treated with 5 μg/ml actinomycin D after 2 h of stimulation.

Given there was no differential effect of costimulation on IL-1β mRNA at 2 h post-treatment (Fig. 3*A*), yet a synergistic effect of the methylated flavonol on TLR2-induced IL-1β protein production was clearly evident at 6 h post-treatment (Fig. 2*A*), we extended our analysis of IL-1β gene expression over an extended time course. From 4 h onwards, we observed significant differences in the effects of each flavonol (Fig. 5*A*). In particular, costimulation with quercetin-3,4′-dimethylether led to the highest accumulation of IL-1β mRNA, 3-fold higher than that observed at the peak of the response to Pam3CSK4 alone. Quercetin-3-methylether had a similar quantitative effect as the dimethylated flavonol when measured at 4 h, but thereafter the levels of mRNA declined. In contrast, costimulation with casticin did not increase the maximal levels of mRNA accumulated beyond those observed for Pam3CSK4 treated cells, but the presence of the flavonol did lead to a significantly sustained response, with the higher levels of IL-1β mRNA persisting up to 24 h, the final time point assayed (Fig. 5*A*).

These different effects of the three flavonols on IL-1β gene expression from 6 h onwards are entirely consistent with their effects on the secretion of IL-1β protein over the extended time course (Fig. 2). Importantly, when the steady-state accumulation of TNF mRNA, which is known to be up-regulated upon TLR2 activation (24), was analyzed following Pam3CSK4 stimulation in the presence or absence of methylated flavonols, the kinetics of TNF mRNA accumulation were near identical (Fig. 5*B*), indicating that the effect of 3-*O*-methylated flavonols was specific to IL-1β. Furthermore, the differential cytokine response of the cells does not arise through a general dosage effect of methyl groups on the flavonol scaffold but rather, reflects an impact of regiospecific methylation.

To determine whether the increase in steady-state levels of IL-1β mRNA observed in costimulated cells was a result of increased mRNA stability, THP-1 cells were stimulated for 2 h and then treated with the transcription inhibitor actinomycin D. In cells treated with actinomycin D, IL-1β mRNA declined to basal levels with the same kinetics, irrespective of whether the cells were treated with Pam3CSK4 alone or costimulated with the methylated flavonols (Fig. 5*C*). This result suggests that the methylated flavonols maintained the ongoing transcription of the IL-1β gene, once that process had been initiated by TLR2 engagement, rather than having an effect on mRNA stability.

**FIGURE 6.**
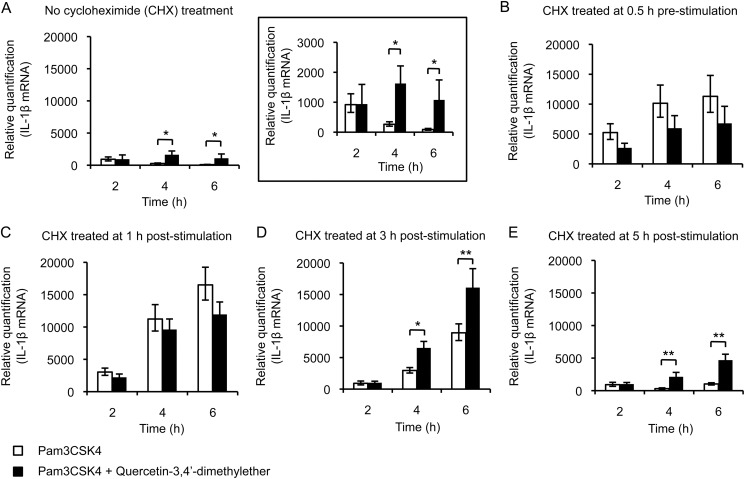
**THP-1 cells treated with cycloheximide show super-induction of IL-1β gene transcription after stimulation with Pam3CSK4 alone or costimulation with quercetin-3,4**′**-dimethylether.** Real-time qPCR analysis of steady-state IL-1β mRNA levels in cells stimulated with Pam3CSK4 alone or costimulated with 10 μm quercetin-3,4′-dimethylether over time. Cells were treated without cycloheximide (*A*, detailed in the *inset*), with 10 μg/ml cycloheximide 30 min prior to stimulation (*B*), or at 1 h (*C*), 3 h (*D*), and 5 h (*E*) post-stimulation. *, *p* < 0.05, **, *p* < 0.01.

To determine whether protein synthesis was required for the flavonols to exert their synergistic effects, THP-1 cells were treated with the translation inhibitor cycloheximide prior to or after their stimulation (Fig. 6). During the first 2 h, pre-treatment with cycloheximide led to enhanced levels of IL-1β mRNA whether the cells were treated with Pam3CSK4 alone, or with Pam3CSK4 and quercetin-3,4′-dimethylether (Fig. 6*B*). A similar super-induction has previously been reported in many studies and is thought to be due to cycloheximide suppression of the resynthesis of NF-κB repressor IκB-α (28, 29). Cells treated with cycloheximide at 1 h post-stimulation showed a similar super-induction effect to that of cycloheximide pretreatment (Fig. 6*C*). Interestingly, the super-induction of IL-1β mRNA was lower in the cells treated with cycloheximide at 3 h post-stimulation of Pam3CSK4 alone, (Fig. 6*D*), and was even lower in those treated with cycloheximide at 5 h post-stimulation of Pam3CSK4 alone (Fig. 6*E*). In contrast, the super-induction of IL-1β mRNA was again observed in the Pam3CSK4 and quercetin-3,4′-dimethylether costimulated cells treated with cycloheximide at 3 h and 5 h post-stimulation (Fig. 6, *D* and *E*). These results suggest that the synergistic effect of the methylated flavonol in up-regulating the transcription of IL-1β from 2 h post-stimulation occurs via a mechanism that requires *de novo* protein synthesis.

## DISCUSSION

TLR signaling pathways are centrally important to the regulation of innate immunity and apoptosis. Exploring the impact of natural products on these pathways provides a useful means of understanding the relationship between diet, inflammation, and cancer. Our study demonstrates that regiospecific modification to a natural product scaffold found commonly in fruits and vegetables has profound effects on its ability to modulate TLR2 signaling. Methylation at different sites on the flavonol influenced the ability of the scaffold to enhance IL-1β production following TLR2 activation, with activity displayed only by the 3-methoxy flavonols casticin, quercetin-3-methylether and quercetin-3,4′-dimethylether (Fig. 1). The results provide new insights into the bioactivities of these natural products and how they may be developed as novel immunomodulators.

**FIGURE 7.**
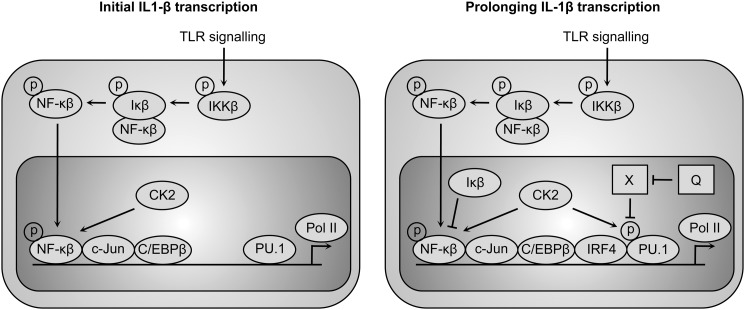
**Regulation of IL-1β gene transcription after TLR activation.** The current model for TLR-dependent IL-1β gene transcriptional activation is regulated in two phases (30). The initial transcription (phase 1) is regulated mainly through NF-κB and the prolonging of transcription (phase 2) involves phosphorylation of PU.1 and recruitment of IRF4 to the promoter region. We hypothesize that, in addition to IκB-α, there is a negative regulator(s) (*X*) switching off the phase 2 transcription, and 3-*O*-methylated quercetin (*Q*) may act as an inhibitor to the negative regulator X. The figure is adapted from Gaestel *et al.* (24) with modifications.

One aspect of our study shows that surprisingly, the effect of the methylated flavonols does not involve inflammasomes, but rather is dependant on transcriptional events. The current model for TLR-dependent transcriptional activation of the IL-1β gene describes a two-phase mechanism of regulation (30). In the first phase, phosphorylated NF-κB binds to the promoter and initiates gene transcription. The binding of NF-κB is maximal at 1 h post-stimulation. In the second phase, beginning ∼2 h post-stimulation, additional transcription factors such as c-Jun and IRF4 are recruited to cooperate with the factor PU.1, which constitutively binds to the promoter and prolongs gene transcription beyond the first phase (Fig. 7) (24, 30). Significantly, our kinetic analysis of steady-state levels of IL-1β mRNA in response to TLR2 signaling and costimulation with 3-*O*-methylated flavonols shows that the flavonols only affect IL-1β gene transcription from 2 h onwards (Fig. 5). Furthermore, we found that the NF-κB phosphorylation profiles from 0–2 h were similar in cells stimulated with Pam3CSK4 alone or costimulated with methylated flavonol (Fig. 3). These observations lead us to conclude that the methylated flavonols affect the second phase of the regulation mechanism, as defined in the model of Zhang *et al.* (30).

Cycloheximide treatment in TLR-activated cells is known to lead to a super-induction of IL-1β gene transcription. This is due to inhibition of the resynthesis of NF-κB repressor IκB-α (28, 29). IκB-α is typically resynthesized 1 h after the initial TLR agonist stimulation, and binds to the activated NF-κB in the nucleus resulting in the inhibition of NF-κB activity and translocation of the protein complex to the cytosol. In our cycloheximide treatment study, super-induction of IL-1β gene transcription was observed in the cells treated with cycloheximide 0.5 h prior to Pam3CSK4 stimulation and 1 h post-stimulation with Pam3CSK4, and to a lesser extent in 3 h post-stimulation with Pam3CSK4. The super-induction effect was not significant in the cells treated with cycloheximide at 5 h post-stimulation with Pam3CSK4 (Fig. 6). The data suggest that the suppression of IL-1β gene transcription requires *de novo* synthesis of negative regulator(s) such as IκB-α to at least 3 h post-stimulation. Interestingly, in the costimulated cells, cycloheximide treatment at 3 h and 5 h post-stimulation led to significantly higher IL-1β mRNA levels compared with the cells stimulated with Pam3CSK4 alone (Fig. 6, *D* and *E*), suggesting that quercetin-3,4′-dimethylether acts as an inhibitor to the negative regulator(s). The target of the methylated quercetin molecules is unlikely to be IκB-α, since IL-1β gene transcription initiation during TLR agonist stimulation shares a common NF-κB signaling pathway with TNF gene transcription initiation (24), and in our study the steady-state TNF mRNA profile in the costimulated cells was found to be similar to that of the cells stimulated with Pam3CSK4 alone (Fig. 5*B*). We therefore hypothesize that the target of quercetin methylether is a negative regulator acting on the second phase of this regulation mechanism, such as that involving in recruitment of IRF4 (Fig. 7).

In contrast to the ability of the methylated flavonols to enhance IL-1β production in our assay system of stimulated THP-1 cells, several earlier studies have shown that flavonoid scaffolds can also inhibit the upstream signaling events that lead to IL-1β gene transcription. These studies have involved a wide range of different mammalian cell types and assay systems (31–43). Thus, for example a number of flavanones, flavones, and flavonols were found to inhibit the activation of NF-κB in cells treated with the TLR4 agonist LPS, and some of those molecules were also found to block the activation of MAPKs (31, 35–37), as well as suppress casein kinase 2 activity and the IRF-4 recruitment to the IL-1β promoter (30).

Flavonols in the diet can be metabolized into methylated forms within epithelial cells of the small intestine, with release both into the bloodstream and also back into the intestinal lumen (44, 45). Methylation of flavonols is also carried out in the liver (46). Thus, the impact of these natural products may not only be limited to events in the intestinal lumen but also systemically throughout the body. This has implications for how these methylated products affect the response of intestinal macrophages and other phagocytic cells to bacterial TLR2 ligands, but also for their effects on other cell types elsewhere. For example, quercetin-3 methylether has been reported to inhibit neutrophil elastase (47), and quercetin-3′-methylether as well as its 4′-isomer inhibit COX-2 production in the human colorectal cancer cell line HCA-7 (48).

In a previous study of methylated flavonols, these molecules were found to induce apoptosis in human tumor cell lines and significantly the 3-methoxy group was found to be the structural feature that determined their anti-proliferative activity (49–52). Given the role of innate signaling in tumorigenesis (53), and our data showing the importance of scaffold methylation on modulation of cytokine production, it is tempting to speculate that at least some of the observed anti-cancer effects of flavonols are related to an ability to fine tune innate immune recognition as well as an ability to affect apoptosis. The precise way in which methylation affects the function of the flavonol scaffold in these systems is yet to be identified.

In summary, our data demonstrating the impact of regiospecific methylation of flavonols on TLR2 signaling, when considered within the wider context of known interactions of innate immunity and apoptosis, offers a new platform for development of pharmaceuticals and nutraceuticals from plant natural products.

## Footnotes

3 The abbreviations used are:

TLR: Toll-like receptor
NOD: nucleotide-binding/oligomerization domain
Ab: antibody.
